# Zika Vaccine Microparticles (MPs)-Loaded Dissolving Microneedles (MNs) Elicit a Significant Immune Response in a Pre-Clinical Murine Model

**DOI:** 10.3390/vaccines11030583

**Published:** 2023-03-03

**Authors:** Akanksha Kale, Devyani Joshi, Ipshita Menon, Priyal Bagwe, Smital Patil, Sharon Vijayanand, Keegan Braz Gomes, Mohammad N. Uddin, Martin J. D’Souza

**Affiliations:** Vaccine Nanotechnology Laboratory, Center for Drug Delivery and Research, Mercer University College of Pharmacy, Atlanta, GA 30341, USA

**Keywords:** Zika, vaccine, microparticles, microneedles

## Abstract

Although the global Zika epidemic in 2015–16 fueled vaccine development efforts, there is no approved Zika vaccine or treatment available to date. Current vaccine platforms in clinical trials are administered via either subcutaneous or intramuscular injections, which are painful and decrease compliance. Therefore, in the present study, we explored Zika vaccine microparticles (MPs)-loaded dissolving microneedles (MNs) with adjuvant MPs encapsulating Alhydrogel^®^ and MPL-A^®^ administered via the transdermal route as a pain-free vaccine strategy. We characterized the MNs for needle length, pore formation, and dissolvability when applied to murine skin. Further, we evaluated the in vivo efficacy of vaccine MPs-loaded MNs with or without adjuvants by measuring the immune response after transdermal immunization. The vaccine MPs-loaded dissolving MNs with adjuvants induced significant IgG, IgG1, and IgG2a titers in immunized mice compared to the untreated control group. After the dosing regimen, the animals were challenged with Zika virus, monitored for seven days, and sacrificed to collect spleen and lymph nodes. The lymphocytes and splenocytes from the immunized mice showed significant expressions of helper (CD4) and cytotoxic (CD8a) cell surface markers compared to the control group. Thus, this study puts forth a ‘proof-of-concept’ for a pain-free transdermal vaccine strategy against Zika.

## 1. Introduction

Zika is a zoonotic disease caused by the Zika virus, which is transmitted to humans primarily through mosquito bites [1]. Although the virus is endemic in developing countries [2], the United States also experienced travel-associated cases of Zika infection. There was a global outbreak of Zika in 2015–16 leading to the declaration of a public health emergency of international concern by the World Health Organization (WHO) [3]. Zika-related research has also been included in the priority R&D list by the WHO [4]. Despite these efforts, there is no approved vaccine or therapy available to combat Zika to date. After infecting an individual, the virus may remain dormant for 14 days, after which symptoms may start emerging [5]. Common symptoms associated with the infection include fever, headache, rash, conjunctivitis, and muscle and body pain, which are treated by non-steroidal anti-inflammatory drugs (NSAIDs). Zika also has the potential to cross the placenta and transfer from a pregnant female to her fetus. This vertical transmission of the Zika virus can result in microcephaly, congenital Zika syndrome, and other congenital birth defects in the newborn [6]. Zika infection also results in an autoimmune disorder called Guillain–Barré Syndrome, which affects the peripheral nervous system [7,8]. It is a syndrome characterized by the degradation of the myelin sheath of nerve fibers, eventually leading to paralysis and death. Such life-threatening events associated with Zika infection highlight the need for a safe and effective vaccine for this virus.

Researchers have explored conventional vaccine platforms, such as inactivated vaccines [9] and live attenuated vaccines, against Zika. Advanced vaccine platforms, such as VLP-based vaccines [10], DNA vaccines [11,12], and mRNA vaccines [13], are also being explored. Most of these vaccines are formulated as solutions for injections. However, data from several studies by our research group show that particulate vaccines induce stronger immune responses than conventional solution-based vaccines. The encapsulation of vaccine antigens in polymeric micro-/nano-particles offers several advantages over solution-based vaccines. It protects the encapsulated antigen from degradation and allows for a sustained release of the antigen, thereby reducing the number of doses [14,15]. It also enables a better uptake and presentation of vaccine antigens by antigen-presenting cells (APCs) [16]. A previous study in our lab showed that inactivated Zika virus encapsulated in poly (lactic-co-glycolic) acid microparticles (MPs) along with Alhydrogel^®^ and monophosphoryl lipid-A (MPL-A^®^) as adjuvants were able to induce a significant immune response compared to an inactivated virus solution in a pre-clinical murine model [14]. In the current study, we went a step further by loading these MPs into dissolving microneedles (MNs) to deliver the vaccine via a pain-free transdermal route.

Traditionally, vaccines are administered either via subcutaneous or intramuscular routes of administration. Invasive injections need trained personnel for administration, generate sharp waste, and are painful, all of which are highly undesirable events. These disadvantages emphasize the need to explore alternative pain-free delivery routes, such as oral, buccal, intranasal, intravaginal, and transdermal routes. The presence of specialized APCs called Langerhans cells, in addition to dermal dendritic cells and resident macrophages in the skin, makes the transdermal route highly conducive for immunization [17]. Langerhans cells play an important role in immune surveillance and further signaling to T cells. Activated T cells are drained into nearby lymph nodes and stimulate B cells to produce antibodies [18]. Additionally, the skin is the primary entry site for the Zika virus, where a blood-feeding mosquito injects the virus into the host. Hamel et. al. showed that dermal fibroblasts, epidermal keratinocytes, and immature dendritic cells are permissive to Zika virus infection [19]. Administering the vaccine at the pathogen entry site leads to the induction of an effective immune response and protection. Thus, the skin is the ideal target site for administering a Zika vaccine.

Previously, our research group explored transdermal vaccine delivery strategies, such as dissolving microneedles (MNs) [15,20] and LASER ablation-mediated delivery [16,21,22], for measles, influenza, COVID-19, and rabies. MNs are micron-sized needles that form microchannels when inserted into the skin, facilitating the entry of vaccine antigens into the skin. Due to their micron length, MNs do not reach the dermal layer of the skin that contains the nerve endings, and, hence, they are painless [23]. Different types of MNs, including metal MNs, hollow MNs, coated MNs, and dissolving MNs, have been previously studied for vaccine delivery. The first two types of MNs are used to create pores in the skin for the application of a liquid formulation. Coated MNs release the cargo after insertion into the skin. Lastly, dissolving MNs dissolve in the skin and release the embedded vaccine cargo. Several polymers, sugars, and their combinations have been explored as matrices for dissolving MNs. An appropriate combination of polymers and sugar is required to achieve MNs with the desired sharpness, mechanical strength, dissolution time, and release profile. A screening study by our research group showed that a combination of sodium hyaluronate and trehalose provides a fast-dissolving matrix for influenza vaccine MPs-loaded MNs [20]. We selected a similar combination for Zika vaccine MPs-loaded dissolving MNs to achieve faster dissolution.

An investigation of a DNA vaccine for Zika administered via intradermal injection followed by electroporation showed promising results in the past [12]. However, Zika vaccine MPs-loaded dissolving MNs have not been previously explored as a vaccine delivery strategy for Zika. Our approach of integrating the advantages of both MPs and MNs provides a proof-of-concept for an effective vaccine strategy against Zika. In the present study, Zika vaccine MPs-loaded dissolving MNs with and without adjuvant MPs (Alhydrogel^®^ and MPL-A^®^) were formulated. MNs were characterized for morphology, needle length, and their ability to form pores in murine skin. Further, the in vivo efficacy of Zika vaccine MPs-loaded dissolving MNs along with adjuvant MPs after transdermal immunization was evaluated by measuring antibody titers in the serum of immunized mice. Finally, the mice were challenged with a live Zika virus, and the cell-mediated immune response was determined by measuring CD4 and CD8a cell surface markers in splenocytes and lymphocytes.

## 2. Materials and Methods

### 2.1. Materials

The Zika virus strain PRVABC59 with a viral titer of 1 × 10^6^ PFU/mL was kindly supplied by Brandy Russell, Centers for Disease Control and Prevention (CDC), Colorado. The polymer poly (D, L-lactide-co-glycolide) 75:25 (PLGA) (Resomer^®^ RG 752 H) was procured from Evonik Corporation (Birmingham, AL, USA). Adjuvants, Alhydrogel^®^ and Monophosphoryl Lipid A (MPL-A^®^), were purchased from InvivoGen (San Diego, CA, USA). Sodium hyaluronate (HA) (100 kDa) was purchased from Lifecore Biomedical (Chaska, MN, USA). Trehalose dihydrate and polyvinyl alcohol (PVA) (Avg Mol Wt. 30,000–70,000) were procured from Sigma-Aldrich (St. Louis, MO, USA). The 10 × 10 array poly dimethyl siloxane (PDMS) microneedle templates were obtained from Micropoint Technologies (Singapore, Singapore). Methylene blue dye was purchased from Fischer Scientific (Hampton, NH, USA). Swiss Webster mice (6–8 weeks old, male) for in vivo efficacy evaluation were purchased from Charles River Laboratories (Wilmington, MA, USA). Goat anti-mouse secondary IgG, IgG2a, and IgG1 conjugated to Horseradish Peroxidase (HRP) were purchased from Invitrogen™ (Rockford, IL, USA). Allophycocyanin (APC)-labeled anti-mouse CD4 antibody and fluorescein isothiocyanate (FITC)-labeled anti-mouse CD8a antibody were purchased from Invitrogen™, Thermo Fisher Scientific (Waltham, MA, USA).

### 2.2. Formulation of Zika Vaccine MPs-Loaded Dissolving MNs

Formulation, physiochemical characterization, and in vitro immunogenicity and cytotoxicity evaluations of Zika vaccine MPs with and without adjuvant MPs (Alhydrogel^®^ and MPL-A^®^) have been published earlier [14]. Briefly, the inactivated Zika virus strain PRVABC59 as an antigen was encapsulated in PLGA vaccine MPs using the double emulsion–solvent evaporation method. The primary emulsion was obtained by emulsifying the antigen in a 2% PLGA solution in dichloromethane (DCM) using an Omni TH_Q_ probe homogenizer (Kennesaw, GA, USA). Next, the double emulsion was made by emulsifying the primary emulsion in a 0.1% polyvinyl alcohol (PVA, MW 30,000–70,000, Sigma-Aldrich, Burlington, MA, USA) solution. To reduce the particle size, the formulation was passed through a Nano DeBEE high-pressure homogenizer for six cycles. Next, to evaporate DCM, the formulation was kept under constant stirring for 4 h. The final formulation was subjected to ultracentrifugation and lyophilization using a Labconco™ FreeZone Triad benchtop freeze dryer. The adjuvants, Alhydrogel^®^ and MPL-A^®^, were encapsulated in PLGA MPs following a similar method.

Zika vaccine MPs-loaded dissolving MNs were formulated by using the spin-cast method as described previously [15]. Briefly, an HA-trehalose gel was prepared by combining 10% *w/v* HA and 5% *w/v* trehalose in deionized water. A pre-weighed vaccine (equivalent to (20 µg) and adjuvant MPs (equivalent to 50 µg Alhydrogel^®^ and 5 µg MPL-A^®^) were dispersed into the hyaluronic acid–trehalose gel. Next, 50 mg of gel was added to each pre-weighed silicone mold template (10 × 10 array) and centrifuged at 4000 rpm for 15 min at 15 °C to form the needles. The MNs were allowed to dry overnight in the molds in a desiccator at room temperature, and a layer of 40% PVA was added the next day as a backing layer and allowed to dry. The dried microneedles were carefully removed from the templates. Blank MNs were formulated with blank MPs without any antigen, while inactivated Zika solution-loaded MNs were formulated with an antigen dose equivalent to that of vaccine MPs-loaded MNs.

### 2.3. Morphology of MNs

The morphology of the MPs-loaded MNs was observed using a Phenom™ benchtop scanning electron microscope (SEM) (Phoenix, AZ, USA). To capture images, the MN patch was placed on a metal pin stub using carbon conductive double-coated PELCO Image Tabs™ (Ted Pella Inc., Redding, CA, USA). Images were captured at an acceleration voltage of 5 kV and magnifications of 205×, 225×, and 400×. Needle length was measured by using the scaling feature of the microscope.

### 2.4. Ability of MNs to Form Pores in Murine Skin

The ability of MNs to form pores in ex vivo murine skin was evaluated using methylene blue staining. The skin was sourced from animals that served as non-treated controls in other experiments after euthanizing the animals at the end of the investigations. It was ensured that intact skin physiology was maintained. To facilitate the MN application and observation of pore formation, the hair on the skin samples was removed using a commercial depilatory cream. After treatment with the depilatory cream, the skin samples were washed gently with water and wiped dry. The murine skin was placed on a flat stack of paper towels. MNs were applied to the murine skin for a duration of ten minutes. After treating the skin with MNs, methylene blue dye solution (0.1% *w*/*v*) was applied for five minutes. Next, the skin was cleaned with alcohol swabs and Kimwipes to remove the excess dye, and images were captured using a Plugable USB digital microscope.

### 2.5. In Vivo Study Design

**Figure 1:**
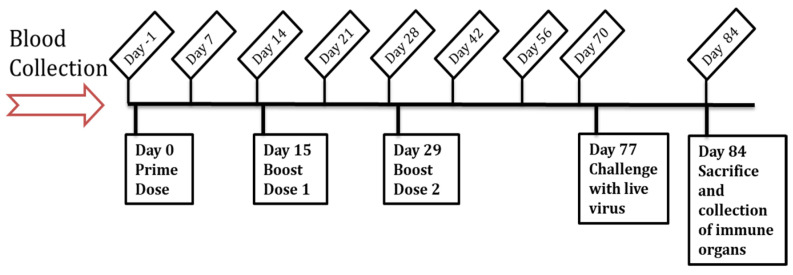
Timeline of the in vivo assessment of Zika vaccine MPs-loaded dissolving MNs.

The in vivo efficacy of the Zika vaccine MPs-loaded MNs with or without adjuvant MPs (Alhydrogel^®^ and MPL-A^®^) was evaluated in 6–8-week-old, male Swiss Webster mice (Charles River Laboratories, Wilmington, MA, USA). All animal experiments were performed in accordance with the IUCAC protocol A2004005 approved by Mercer University’s IUCAC. The animals were quarantined for 7 days before the study. The mice were housed in groups of 2–3 in a temperature- and humidity-controlled room under a 12 h light/dark cycle in Mercer University’s Biosafety Level-2 (BSL-2) facility. The animals were allowed access to food (Laboratory Rodent Diet) and water ad libitum during experimental procedures. Table 1 describes the groups of animals involved in the in vivo efficacy evaluation of the Zika vaccine MPs-loaded dissolving MNs. The animals were assigned to these groups in a random order, with n = 5 per group. The timeline of the in vivo studies is shown in Figure 1. The vaccine antigen dose/mouse was 20 µg of inactivated Zika virus, the Alhydrogel^®^ dose/mouse was 20 µg, and the MPL-A^®^ dose/mouse was 5 µg. The mice received a total of three vaccine doses via the transdermal route. A day before vaccine administration, a 2 × 2 cm^2^ area was cleared of hair from the dorsal side of anesthetized mice using a depilatory cream to facilitate MN administration. After administering the prime dose on day 0, two booster doses were given on day 15 and day 29. Blood samples from the immunized mice were collected at specified times (days 7, 14, 21, 28, 42, 56, and 70 after the prime dose) and centrifuged to collect the serum. The presence of Zika-specific IgG, IgG2a, and IgG1 in the serum samples was determined using an enzyme-linked immunosorbent assay (ELISA). Seven weeks after the last dose, the mice were challenged with 200 µL of a live Zika virus (Strain PRVABC59, 3.65 × 10^5^ PFU/mL) via intraperitoneal injection to assess the expressions of CD4 and CD8a cell surface markers in the immunized mice compared to the control group. The animals were monitored for 7 days for any change in weight by weighing them daily using a digital weighing scale. After seven days of monitoring, the animals were sacrificed, and immune organs, such as the spleen, inguinal, and axillary lymph nodes, were extracted for an evaluation of cell-mediated immune responses.

### 2.6. Measurement of Antibody Titers Using ELISA

To measure the antibody titers, the serum samples were tested using ELISA as described previously [14]. The incubation temperature and time are included in parentheses for every step. The inactivated Zika strain PRVABC59 was used as the coating antigen to coat high-binding polystyrene ELISA plates (Microlon^®^) (50 ng/well at 4 °C, overnight). After the coating step, the plate was blocked with 3% BSA (37 °C, 2 h) to prevent nonspecific binding. Next, the serum samples were diluted to 1:100 dilution with PBS and were added to the ELISA plate (37 °C, 2 h). After the incubation of the serum samples for the designated time, HRP-conjugated goat anti-mouse secondary antibody (either IgG or IgG1 or IgG2a) was added (37 °C, 2 h). 3,3′,5,5′-tetramethylbenzidine (TMB) (BioLegend^®^, San Diego, CA, USA) was used as the substrate. Finally, 0.3 M sulfuric acid was added to stop the reaction. The plates were washed extensively before adding every reagent with 0.1% TWEEN 20 in PBS as a wash buffer. After the final step, the absorbance was measured at 450 nm using a BioTek^®^ Synergy H1 microplate reader (BIO-TEK Instruments, Winooski, VT, USA).

The IgG1:IgG2a ratio was calculated for the groups receiving the vaccine solution, vaccine MPs-loaded MNs, and adjuvanted vaccine MPs-loaded MNs.

### 2.7. Measurement of T-Cell Surface Markers

After the dosing regimen, the animals were challenged with the live Zika virus via an intraperitoneal injection as described in the in vivo study design. Seven days later, the mice were sacrificed, and immune organs, such as the spleen, inguinal, and axillary lymph nodes, were extracted to assess the T-cell responses as described previously [21]. The organs were processed into single-cell suspensions using a 40 µm cell strainer. The spleen samples were treated with an ammonium chloride potassium (ACK) lysis buffer to eliminate red blood cells (RBCs). The cells were centrifuged at 1200 rpm and suspended in Dulbecco’s Modified Eagle Medium (DMEM) supplemented with 70% fetal bovine serum (FBS). The samples were stored at −80 °C with 5% *v/v* dimethyl sulfoxide (DMSO) as a cryoprotectant until further analyses. To assess the expressions of CD4 and CD8a in lymphocytes and splenocytes, the cells were thawed on ice, washed, and resuspended in phosphate buffer saline (PBS). The cell suspensions were stimulated with 5 µg/mL IL-2 overnight, and then they were treated with 100 uL of anti-mouse APC-labeled CD4 and FITC-labeled CD8a antibodies in PBS and incubated for 1 h on ice, protected from light. After the incubation period, the cells were washed to remove excess markers and analyzed using a BD Accuri C6 Plus flow cytometer (BD Bioscience, San Jose, CA, USA).

### 2.8. Statistical Analysis

All the experiments were performed in triplicate unless otherwise specified. The normality of the data was tested by using the Shapiro–Wilk test, and the equality of variances was tested by using the Brown–Forsythe test. For datasets exhibiting normal distribution, the One-Way Analysis of Variance (ANOVA) test with either Tukey’s or Dunnett’s multiple comparison post hoc test was performed. For datasets without normal distribution, a nonparametric Kruskal–Wallis test followed by a post hoc analytical test was performed. To determine statistical significance, a *p*-value of <0.05 was considered. GraphPad Prism version 9.2.0 for Windows (GraphPad Software, San Diego, CA, USA, www.GraphPad.com) was used to conduct the statistical analyses. The datasets are reported as mean ± SEM unless otherwise stated. We have routinely used similar analyses in mouse models. Effect sizes were calculated by using Eta^2^ for ANOVA results.

## 3. Results

### 3.1. Morphology of Dissolving MNs

**Figure 2:**
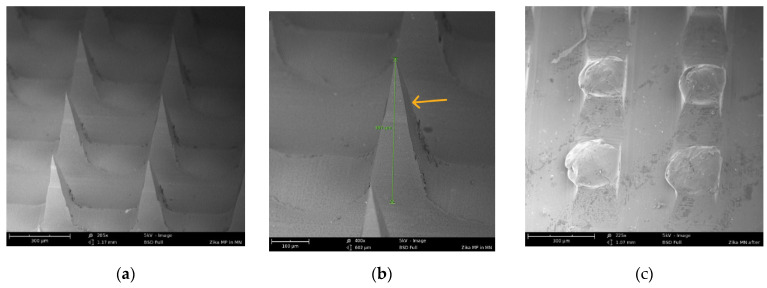
(**a**) SEM image (205×) of pyramid-shaped MNs in the array before application to murine skin. (**b**) SEM image (400×) showing the length (387 um) of one pyramid-shaped microneedle in the array. (**c**) SEM image (225×) of dissolved microneedles in the array after application to murine skin for 10 min.

The morphology of the Zika vaccine MPs-loaded dissolving MNs was observed using a scanning electron microscope. It was observed that pyramid-shaped MNs formed with a sharp tip, and the average length of the MNs was 387 µm, as shown in Figure 2. These MNs dissolved within 10 min of application into the murine skin ex vivo.

### 3.2. Ability of MNs to Form Pores in Murine Skin

**Figure 3:**
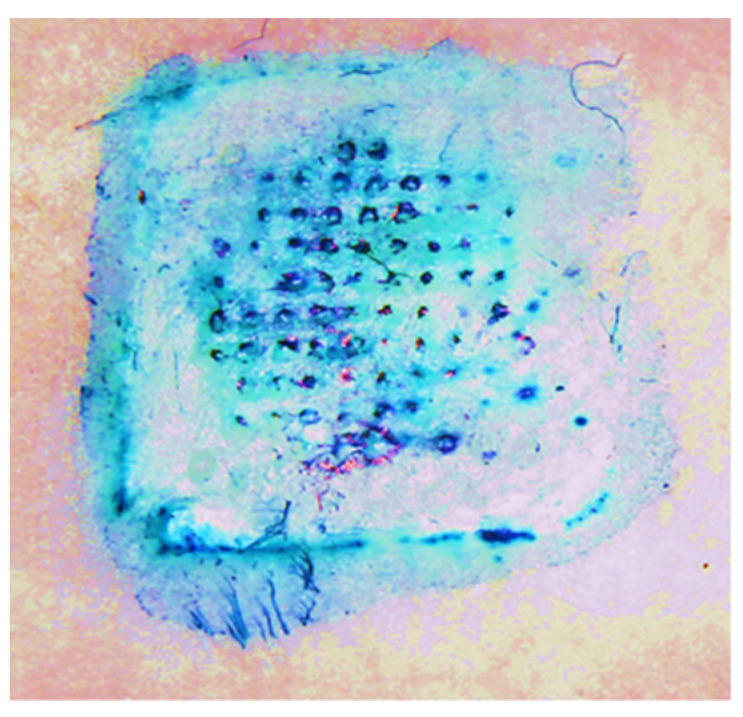
Observation of pores formed by Zika vaccine MPs-loaded dissolving MNs in the murine skin using methylene blue staining.

The ability of the Zika vaccine MPs-loaded dissolving MNs to form pores in the murine skin was observed using methylene blue staining. As shown in Figure 3, the MNs were able to form significant pores in the murine skin.

### 3.3. Measurement of Antibody Titers Using ELISA

The ability of the Zika vaccine MPs-loaded MNs with or without adjuvants to induce the humoral immune response was evaluated in Swiss Webster mice after transdermal immunization.

**Figure 4:**
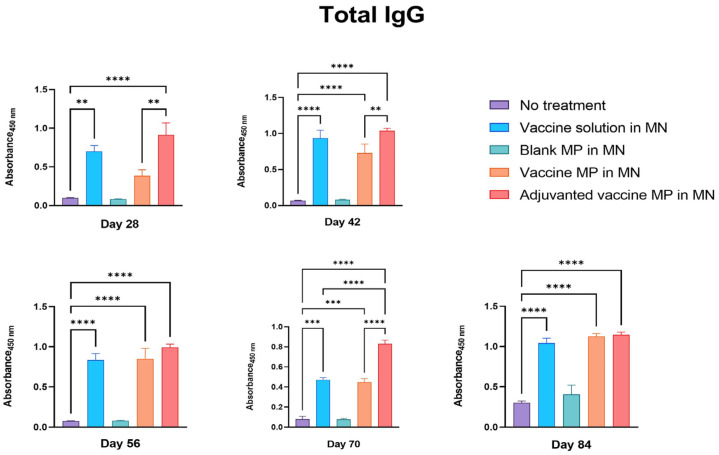
Measurement of Zika-specific total IgG in serum using ELISA. Mice were immunized with one prime dose on day 0 and two booster doses on days 15 and 29 via the transdermal route. Mice receiving vaccine solution, vaccine MPs, and adjuvanted vaccine MPs-loaded MNs produced significantly higher antibody titers than the untreated control group (days 28, 42, 56, 70, and 84). Moreover, the group receiving adjuvanted vaccine MPs showed higher titers than the group receiving vaccine MPs without adjuvants (days 28, 42, 70) (data presented as mean ± SEM, n = 5, One-Way ANOVA test followed by Tukey’s multiple comparison tests; **, *p* < 0.01, ***, *p* < 0.001, ****, *p* < 0.0001).

The groups of mice receiving the Zika vaccine solution, vaccine MPs, and adjuvanted vaccine MPs-loaded MNs produced significant IgG when compared to the control group of untreated mice. Moreover, the mice receiving the adjuvanted vaccine MPs produced significantly higher antibody titers than the mice receiving the vaccine solution and the vaccine MPs. These antibody titers remained higher even post-challenge with the live Zika virus (Figure 4).

**Figure 5:**
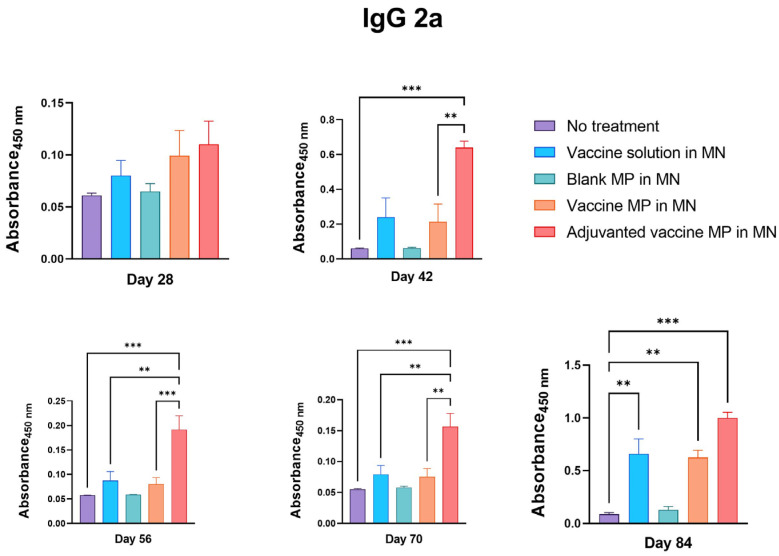
Measurement of Zika-specific IgG2a in serum using ELISA. Mice were immunized via transdermal route with one prime dose on day 0 and two booster doses on days 15 and 29. Mice receiving adjuvanted vaccine MPs induced significantly higher antibody titers than the untreated control group (days 42, 56, 70, and 84). Moreover, the group receiving adjuvanted MNs showed higher titers than the group receiving vaccine MPs without adjuvants (days 42, 56, 70). Seven days after the challenge with live virus, mice receiving vaccine solution, vaccine MPs, and adjuvanted vaccine MPs induced significantly higher antibody titers than the untreated control group (day 84). (data presented as mean ± SEM, n = 5, One-Way ANOVA test followed by Tukey’s multiple comparison test; **, *p* < 0.01, ***, *p* < 0.001).

Further, the serum samples of the immunized mice were tested for IgG2a and IgG1 subtypes. It was observed that the group of mice receiving the adjuvanted vaccine MPs-loaded MNs produced significantly higher IgG2a antibodies than the control group. Moreover, the IgG2a titers in this group were significantly higher than those in the group receiving the vaccine solution and the vaccine MPs without adjuvants (Figure 5).

**Figure 6:**
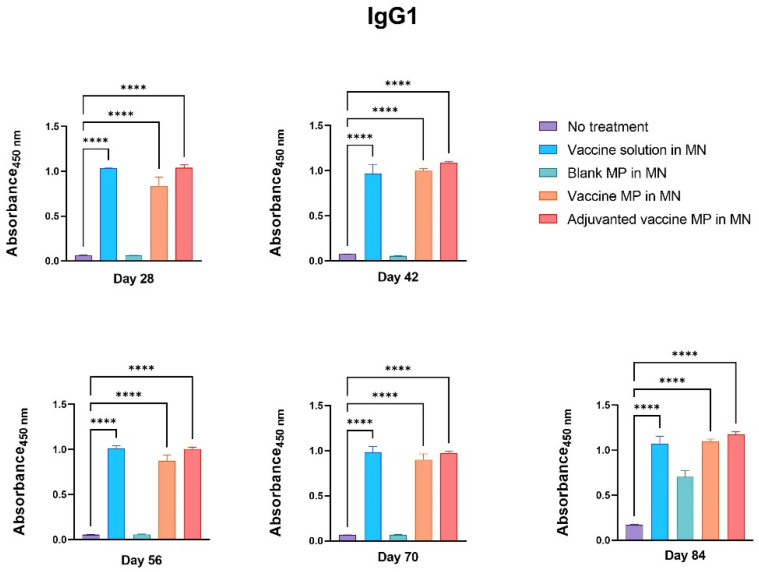
Measurement of Zika-specific IgG1 using ELISA. Mice were immunized via transdermal route with one prime dose on day 0 and two booster doses on days 15 and 29. Mice receiving vaccine solution, vaccine MPs, and adjuvanted vaccine MPs produced significantly higher antibody titers than the untreated control group (days 28, 42, 56, 70, and 84) (data presented as mean ± SEM, n = 5, One-Way ANOVA test followed by Dunnett’s multiple comparison test; ****, *p* < 0.0001).

The groups of mice receiving the vaccine solution, vaccine MPs, and adjuvanted vaccine MPs-loaded MNs produced significantly higher IgG1 titers than the control group (Figure 6).

The group of mice receiving the blank MPs-loaded MNs did not produce any significant antibody titers, indicating that the MP and MN matrices are non-immunogenic (Figure 4, Figure 5 and Figure 6).

**Figure 7:**
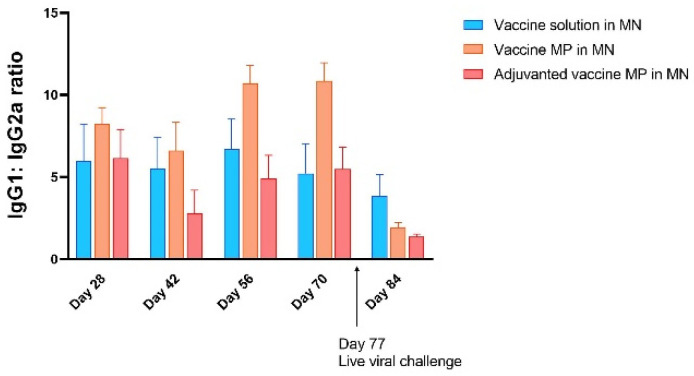
Calculation of IgG1:IgG2a ratio. A higher IgG1:IgG2a ratio in the group receiving vaccine MPs showed a Th2-oriented immune response after immunization, while a lower IgG1:IgG2a ratio in the group receiving adjuvanted vaccine MPs indicated a Th1-oriented immune response among the groups under consideration. After the live viral challenge, the ratio decreased further, indicating a shift towards a Th1-oriented immune response (data presented as mean ± SEM, n = 5).

Next, the IgG1:IgG2a ratio was calculated for the groups receiving the vaccine solution, vaccine MPs, and adjuvanted vaccine MPs-loaded MNs using the absorbance values. A higher IgG1:IgG2a ratio was observed for the group receiving the vaccine MPs-loaded MNs, indicating a Th2-oriented immune response, followed by the group receiving the vaccine solution-loaded MNs. A lower IgG1:IgG2a ratio was observed in the group receiving the adjuvanted vaccine MPs-loaded MNs, indicating a Th1-oriented immune response. This can be attributed to the presence of adjuvant MPL-A, which is known to induce a Th1-mediated immune response. However, after the live viral challenge, the IgG1:IgG2a ratio decreased for the groups receiving the vaccine MPs and further for the group receiving the adjuvanted vaccine MPs (Figure 7).

### 3.4. Measurement of T-Cell Surface Markers

**Figure 8:**
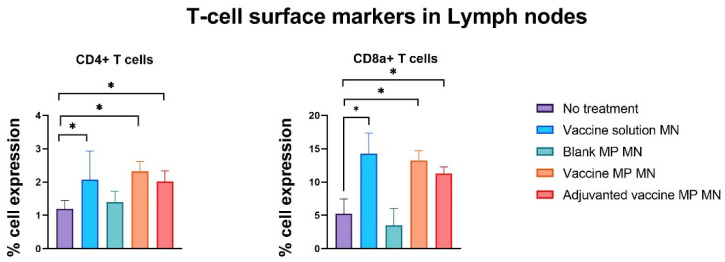
Assessment of T-cell surface markers in lymph nodes of immunized mice using flow cytometry. Mice were immunized via transdermal route with one prime dose on day 0 and two booster doses on days 15 and 29. Seven days post-challenge with live Zika virus, mice were sacrificed, and the lymph nodes were extracted, processed into single-cell suspensions, and analyzed for the presence of T-cell surface markers. Samples from mice receiving vaccine solution, vaccine MPs, and adjuvanted vaccine MPs showed significantly higher expressions of CD4 and CD8a cell surface markers than the control group (data presented as mean ± SEM, n = 5, One-Way ANOVA test followed by Dunnett’s multiple comparison test; *, *p* < 0.05).

**Figure 9:**
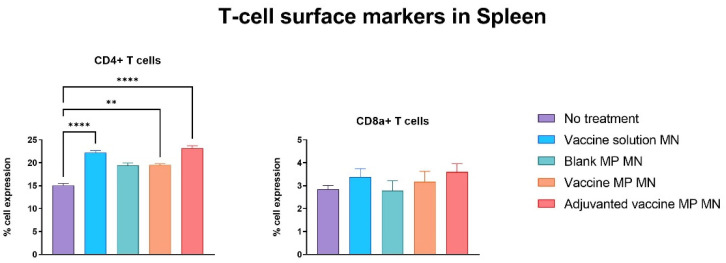
Assessment of T-cell surface markers in the spleen of immunized mice using flow cytometry. Mice were immunized via transdermal route with one prime dose on day 0 and two booster doses on days 15 and 29. Seven days post-challenge with live Zika virus, mice were sacrificed, and the spleens were extracted, processed into single-cell suspensions, and analyzed for the presence of T-cell surface markers. Samples from mice receiving vaccine solution, vaccine MPs, and adjuvanted vaccine MPs showed significantly higher expressions of CD4 cell surface markers than the control group. Expression of CD8a T-cell surface marker was not significant in spleen samples (data presented as mean ± SEM, n = 5, One-Way ANOVA test followed by Dunnett’s multiple comparison test; **, *p* < 0.01, ****, *p* < 0.0001).

The lymph nodes and spleen were extracted from the immunized mice seven days post-challenge with the live Zika virus. The single-cell suspensions of these markers were assessed for T-cell surface markers using flow cytometry. The lymphocytes of the mice immunized with the vaccine solution, vaccine MPs, and adjuvanted vaccine MPs-loaded MNs showed significantly higher expressions of CD4 and CD8a T-cell surface markers when compared to the control group of untreated mice (Figure 8). The splenocytes of the mice immunized with the vaccine solution, vaccine MPs, and adjuvanted vaccine MPs-loaded MNs showed significantly higher expressions of CD4 T-cell surface markers when compared to the control group of untreated mice. The expression of CD8a T-cell surface markers was not significant in these groups (Figure 9). (Flow cytometry plots are provided as Supplementary Data (Figures S1 and S2)).

## 4. Discussion

We formulated Zika vaccine MPs-loaded dissolving MNs along with adjuvant (Alhydrogel^®^ and MPL-A^®^) MPs, and we characterized and evaluated them for in vivo efficacy by measuring antibody-mediated and cell-mediated immune responses in a pre-clinical murine model after transdermal immunization. We found that the Zika vaccine MPs-loaded dissolving MNs along with adjuvant (Alhydrogel^®^ and MPL-A^®^) MPs induced a significant humoral, as well as a cell-mediated, immune response. Moreover, the antibody responses were higher in the group receiving the adjuvanted vaccine MPs than in the groups receiving just the inactivated vaccine solution or vaccine MPs. Previously, we showed that the microparticulate vaccine encapsulating an inactivated Zika virus along with adjuvants induces a stronger immune response than the inactivated solution-based vaccine after intramuscular (IM) immunization [14]. However, IM injections need trained personnel, generate sharp waste, are painful, and reduce patient compliance. To overcome the limitations of invasive vaccine administration routes, we explored pain-free dissolving MNs administered via the transdermal route as a vaccine strategy against Zika. Dissolving MNs enable the self-administration of vaccines, do not produce sharp waste, and improve patient compliance due to their painless delivery [24]. Additionally, the skin is the principal site for Zika entry, where a blood-feeding mosquito injects the virus, and different types of skin cells are susceptible to Zika viral invasion and replication [19]. Thus, transdermal immunization against Zika will provide an immune defense at the pathogen entry site.

A combination of hyaluronic acid (HA) and trehalose was selected to obtain MNs with the desirable mechanical strength and dissolvability [15,21]. As the name suggests, dissolving MNs dissolve in the skin, creating hydro channels and releasing the cargo, which is taken up by different cells in the skin. Skin is rich in antigen-presenting cells (APCs) called Langerhans cells, dermal dendritic cells, and macrophages, which take up vaccine antigens, process them, and drain into nearby lymph nodes, initiating the cascade of the immune response. Previously, our research group showed that vaccine MPs administered via the transdermal route induce a stronger immune response [15,16,20,25], which was also observed in the present study. Our approach of integrating the advantages of microparticulate vaccines with pain-free transdermal administration provides a promising vaccine strategy against Zika. This is significant for vaccine distribution to developing countries where Zika infections are endemic [2].

It is a well-known fact that adjuvants are immunostimulatory molecules that enhance the immune response of vaccine antigens. Alhydrogel^®^ is the most commonly used adjuvant in vaccine formulations. It is an aluminum hydroxide gel that recruits APCs at the vaccine administration site, promotes antigen uptake, and is involved in stimulating the antibody-mediated Th2 response. However, MPL-A^®^ leads to enhanced phagocytosis by APCs and macrophages and enhances the expression of MHC molecules necessary for antigen presentation to naive T cells. It is a TLR4 agonist that induces the cell-mediated Th1 response by interacting with APCs and enhancing the release of cytokines, such as IL-2 and TNF-α [26]. Thus, these two adjuvants complement one another’s mechanism in inducing a balanced immune response. A combination of aluminum salt and MPL-A^®^ has been approved by the FDA as an adjuvant system (AS04) for use in human vaccines. A combination of alum and MPL-A^®^ in a subunit Zika vaccine induced robust antibody and cellular immune responses and isotype-switched antibody responses, and it showed improved protection against lethal Zika viral challenge in a murine model [27,28]. Our data also showed a similar trend of enhanced antibody levels and increased expression of CD4+ CD8a+ in mice immunized with the adjuvanted vaccine MPs-loaded dissolving MNs.

The induction of pathogen-specific antibodies is one of the criteria for any vaccine to be effective. The Zika vaccine MPs-loaded MNs with and without adjuvants were able to induce significantly higher Zika-specific IgG, indicating the efficacy of the transdermal vaccine. Different subtypes of IgGs help in combating the pathogen via various mechanisms. The IgG2a subtype of antibodies clears the infection via a cell-mediated Th1 immune response [29,30]. Th1 cells play a critical role in the stimulation of natural killer (NK) cells, macrophages, and cytotoxic T cells and in the secretion of cytokines, such as IL-12 and IFN-γ, to eliminate intracellular viruses. Thus, measuring the IgG2a subtype is one of the methods used to check the Th1-mediated immune response. However, IgG1 is the most abundant IgG subtype playing a critical role in the antibody-mediated Th2 immune response against viral pathogens. It is also important in clearing pathogens via complement-dependent cytotoxicity (CDC) and antibody-dependent cell-mediated cytotoxicity (ADCC) mechanisms [31]. Thus, measuring IgG1 titers is one of the methods used to characterize the Th2-mediated immune response. Balanced Th1- and Th2-mediated immune responses are essential for a vaccine to be effective. Our data show that the Zika vaccine MPs-loaded MNs with and without adjuvants were able to induce both IgG1 and IgG2a subtypes. Further, the IgG1: IgG2a ratio calculation showed that the microparticulate vaccine induced the Th2-mediated immune response after immunization. However, after the viral challenge, the lower IgG1: IgG2a ratio indicated a more Th1-oriented immune response. This highlights the potential of transdermal Zika vaccine MPs to induce both Th1- and Th2-mediated responses.

No significant weight loss was observed in the animals after the live virus challenge, as Swiss Webster mice are not susceptible to viremia. However, the live virus challenge allowed us to determine the T-cell-mediated immune response. In a different cohort of T cells, CD4+ cells are helper T cells that orchestrate the immune response by stimulating macrophages, CD8+ T cells, and B cells. Past studies have shown that CD4+ T cells are necessary for the protection of mice against lethal viral infection. A lack of CD4+ can increase the propensity for neurological consequences associated with the Zika virus [32]. Our data show that the mice immunized with the Zika vaccine MPs-loaded MNs with and without adjuvants showed an enhanced expression of helper CD4 cell surface markers in lymphocytes and splenocytes post-challenge. CD8+ T cells are essential in combating the pathogen by secreting various cytokines, such as IL-4 and IFN-γ, and directly killing the already infected cells. The presence of naïve or Zika immune CD8+ T cells is another correlate of protection against Zika [33]. Our data show that the mice immunized with the Zika vaccine MPs-loaded MNs with and without adjuvants showed an enhanced expression of cytotoxic CD8a cell surface markers in lymphocytes post-challenge. Thus, our novel transdermal vaccine has the potential to induce a cell-mediated immune response. This can be attributed to the Langerhans cells present in the skin that take up the antigen and stimulate the immune response by delivering the antigen to nearby lymph nodes [15,16,17,18,22,34]. However, in addition to CD4/CD8a expression, a detailed analysis of the cytokine profile is also necessary to supplement the cell-mediated immune response.

The blank MPs-loaded MNs did not induce any significant antibody titers or expressions of cell surface markers in lymphocytes. Although there was a higher expression of cell surface markers in splenocytes, it was not statistically significant. This confirmed that the MP and MN matrices were not immunogenic and that the immune response was purely due to the antigen embedded in the delivery system.

However, we acknowledge that the robustness of the immune response needs to be validated by measuring end-point antibody titers and Zika-specific T-cell responses.

Thus, this exploratory study puts forth a ‘proof-of-concept’ for a potential transdermal Zika vaccine. In future studies, we aim to investigate the memory response after transdermal immunization induced by the novel Zika vaccine MPs-loaded dissolving MNs. Follow-up studies will also include measurements of neutralizing antibodies and reductions in viral titers in the serum of immunized mice as correlates of protection after challenge with live virus. Additionally, we also plan to test if this vaccine can induce cross-protection by challenging the mice with different strains of Zika. Antibody-dependent enhancement (ADE) of infection is an important factor of consideration for the development of a vaccine against a flavivirus. Future studies also have the scope to address this factor by challenging mice immunized with the Zika vaccine with another flavivirus.

## 5. Conclusions

In the present study, Zika vaccine MPs-loaded MNs with and without adjuvant MPs were successfully formulated. An in vivo efficacy evaluation after transdermal immunization in mice demonstrated that the vaccine MPs-loaded MNs with and without adjuvants were able to induce significant antibody levels, as well as enhance the expression of CD4 and CD8a cell surface markers in splenocytes and lymphocytes. The induction of IgG2a and IgG1 subtypes and helper (CD4+) and cytotoxic (CD8a+) T-cell surface markers showed that this transdermal vaccine has the potential to induce both Th1- and Th2-mediated immune responses. Thus, this study established the feasibility of a potential transdermal vaccine for Zika.

## Figures and Tables

**Table 1:** Description of groups of mice in the in vivo efficacy evaluation of Zika vaccine MPs-loaded dissolving MNs (n = 5 per group).

Group No.	Description of Treatment
1	No treatment
2	Zika vaccine solution-loaded MNs
3	Blank MPs-loaded MNs
4	Zika vaccine MPs-loaded MNs
5	Zika + adjuvant MPs-loaded MNs

## Data Availability

Data will be made available upon reasonable request.

## Supplementary Materials

The following supporting information can be downloaded at: https://www.mdpi.com/article/10.3390/vaccines11030583/s1, Figure S1: Evaluation of expression of CD4+CD8+ cell surface markers in splenocytes; Figure S2: Evaluation of expression of CD4+CD8+ cell surface markers in lymphocytes.
