# Supplementary material for: Zika Vaccine Microparticles (MPs)-Loaded Dissolving Microneedles (MNs) Elicit a Significant Immune Response in a Pre-Clinical Murine Model
Source: Vaccines (Basel). 2023 Mar 3;11(3):583. doi: 10.3390/vaccines11030583 (PMC10056879; doi:10.3390/vaccines11030583)
Supplement: Supplementary file 1 [file vaccines-11-00583-s001.zip › vaccines-2199701-supplementary.pdf]

Supplementary data

The process of selection of cells: First live cells were selected. Then FITC and APC positive cells were selected.

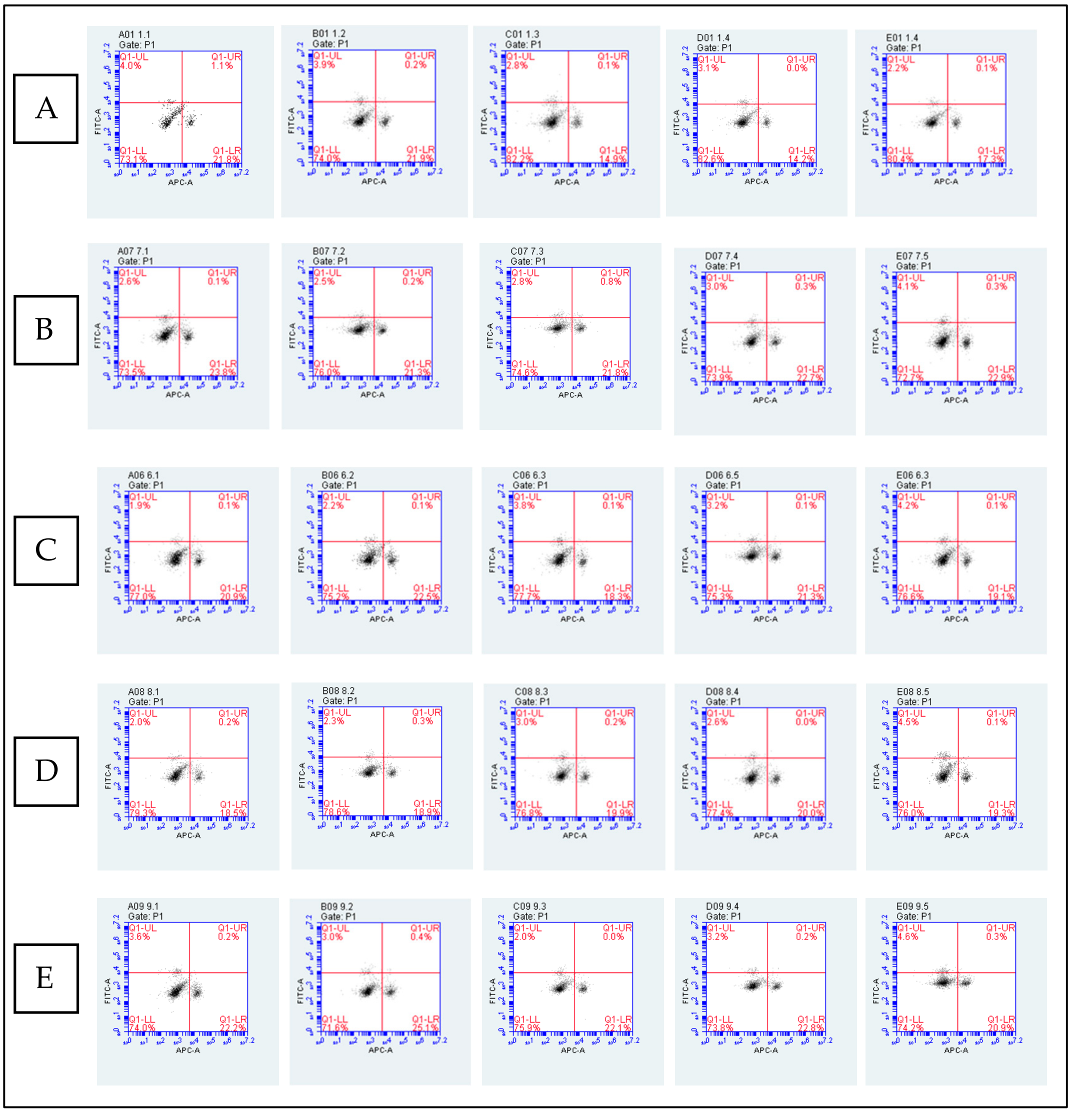

Figure S1: Evaluation of expression of CD4+CD8+ cell surface markers in splenocytes. Panel A represents no treatment group, panel B represents group treated with inactivated Zika vaccine solution-loaded MNs, panel C represents group treated with blank MP-loaded MNs, panel D represents group treated with Zika vaccine MP-loaded MNs, panel E represents adjuvanted (Alyhdrogel + MPL-A) Zika vaccine MP-loaded MNs. APC-conjugated anti-mouse CD4 antibody and FITC-conjugated anti-mouse CD8a antibody were used to stain the cells.

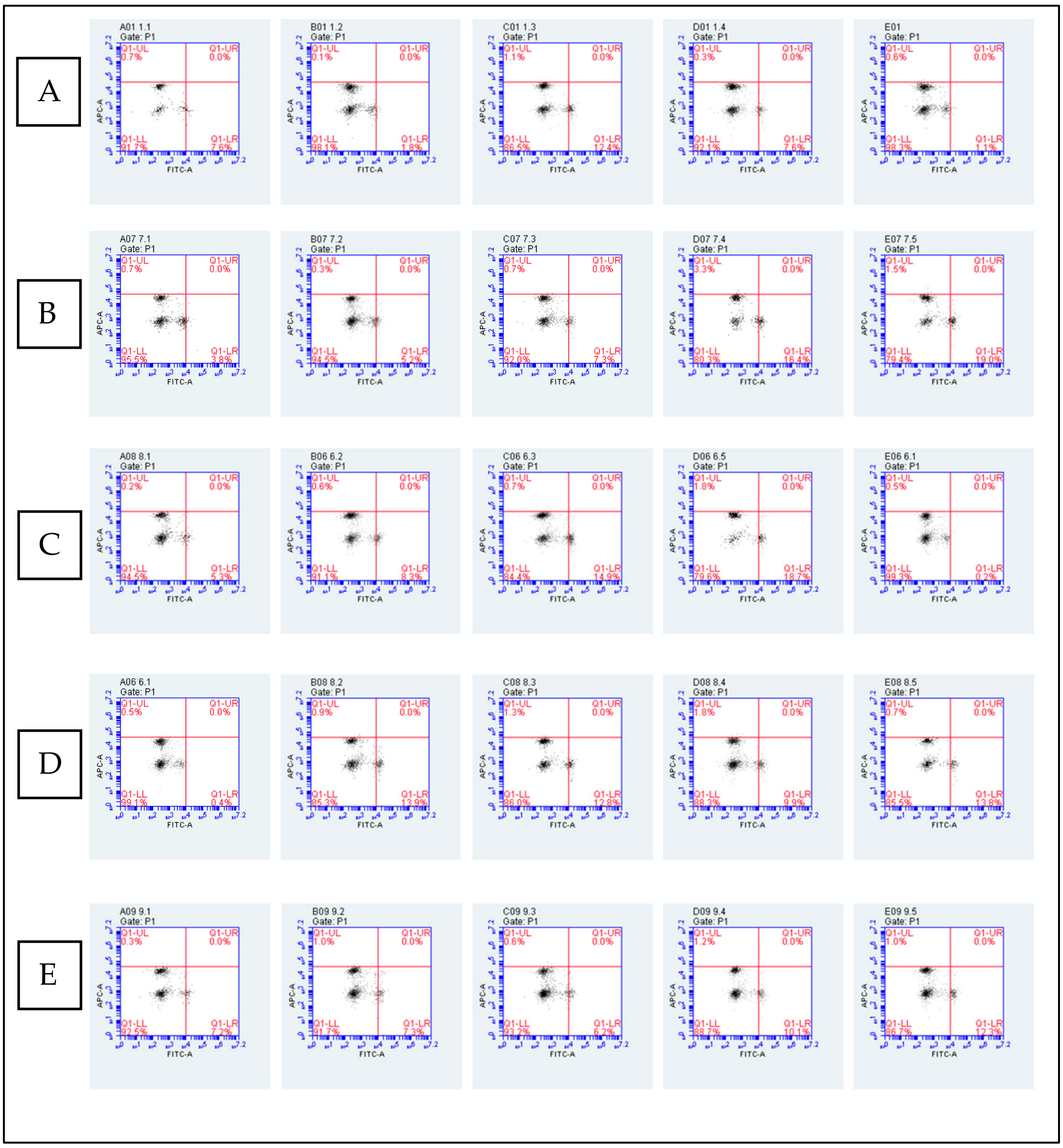

Figure S2: Evaluation of expression of CD4+CD8+ cell surface markers in lymphocytes. Panel A represents no treatment group, panel B represents group treated with inactivated Zika vaccine solution-loaded MNs, panel C represents group treated with blank MP-loaded MNs, panel D represents group treated with Zika vaccine MP-loaded MNs, panel E represents adjuvanted (Alyhdrogel + MPL-A) Zika vaccine MP-loaded MNs. APC-conjugated anti-mouse CD4 antibody and FITC-conjugated anti-mouse CD8a antibody were used to stain the cells.
